# Mutations in gonadotropin-releasing hormone signaling pathway in two nIHH patients with successful pregnancy outcomes

**DOI:** 10.1186/s12958-016-0183-8

**Published:** 2016-08-20

**Authors:** Nikolay Zernov, Mikhail Skoblov, Ancha Baranova, Konstantin Boyarsky

**Affiliations:** 1 Federal State Budgetary Institution “Research Centre for Medical Genetics”, Moskvorechie 1, Moscow, Russia; 2 The Moscow Institute of Physics and Technology, Dolgoprudny, 141700 Moscow Region, Russia; 3 The Russian National Research Medical University named after N.I.Pirogov (RNRMU), Ostrovityanova 1, Moscow, 117997 Russia; 4 Center for the Study of Chronic Metabolic and Rare Diseases, School of Systems Biology, College of Science, George Mason University, 4400 University Dr David King Hall MSN3 E1, Fairfax, VA USA; 5 Atlas Biomed Group, 31 Malaya Nikitskaya Str, Bldg. 1, Moscow, 123317 Russia; 6 Center of Human Reproduction “Genesis”, St. Petersburg, Russia; 7 Department of Obstetrics and Gynecology, State Pediatric Medical University, St. Petersburg, Russia

## Abstract

**Background:**

Anomalous levels of gonadotropin-releasing hormone (GnRH) secretion result in a variety of reproductive phenotypes associated with infertility or subfertility. The normosmic isolated hypogonadotropic hypogonadism (nIHH) is due to a failure of either GnRH pulsatile secretion in hypothalamus or its reception in pituitary. The spectrum of nIHH-associated alterations continues to expand, especially when additional ethnic populations are investigated. The aim of this study was to uncover genetic causes for nIHH in patients of Russian origin.

**Methods:**

For two nIHH patients referred to infertility clinic, both exons and promoter sequences of 6 GnRH signaling genes were sequenced.

**Results:**

Patient 1 was a compound heterozygote for mutations in GnRH and its receptor encoding genes, while in Patient 2 GnRHR mutations were found in homozygous state. In both patients, the coding frame of GnRHR gene harbored missense-mutation Arg139His previously described as founder mutation in Polish and Brazilan patients. IVF/ET treatments were successful, with phenotypically healthy offsprings delivered.

**Conclusion:**

Polish founder mutation Arg139His in GnRHR was found in two nIHH patients originating from Western region of Russia. Common variant of GnRH-encoding gene, Trp16Ser, could possibly contribute to reproductive phenotypes in patients with heterozygous mutations of other GnRH signaling pathway genes.

**Electronic supplementary material:**

The online version of this article (doi:10.1186/s12958-016-0183-8) contains supplementary material, which is available to authorized users.

## Introduction

Despite the latest increase in the volume of relevant publications, the majority of genetic variants influencing sexual development and fertility remain either unknown or poorly characterized in a majority of populations. The secretion of gonadotropin-releasing hormone (GnRH) is essential for the acquisition and maintenance of reproductive competence in mammals [1]. Importantly, anomalous levels of GnRH secretion result in a variety of reproductive phenotypes associated with infertility or subfertility.

Isolated hypogonadotropic hypogonadism, or isolated gonadotropin-releasing deficiency, is relatively rare genetic disease with an occurrence of about 1–10 cases per 100 000 births. In patients with isolated hypogonadotropic hypogonadism, serum concentrations of FSH and LH are low, while the levels of sex steroid are lacking and the puberty is delayed in absence of any other pituitary deficiencies. In about 60 % of these patients, the sense of smell is also impaired, producing either hyposmia or anosmia. The latter condition is called Kallmann syndrome (KS). Current classification of the KS includes 6 subtypes. X-linked KS (type 1) is associated with *KAL1* mutation, while KS subtypes 2,3,4,5 and 6 are linked to the mutations in genes *FGFR1, PROKR2, PROK2, CHD7* and *FGF8*, respectively [2, 3]. Patients with *KAL1* mutation may also have associated disorders of a neurological or urogenital nature, thus, demonstrating more severe phenotype [1–3].

In the remaining 40 % patients, the sense of smell is unchanged. The normosmic isolated hypogonadotropic hypogonadism (nIHH) is due to a failure of either gonadotropin-releasing hormone (GnRH) pulsatile secretion in hypothalamus or its reception in pituitary. In nIHH patients, causal mutations were identified in genes *GnRH1, GnRHR, KISS1, KISS1R, TAC3* and T*AC3R* [2]. Importantly, the spectrum of nIHH-associated alterations continues to expand, especially when additional ethnic populations are investigated.

Here we present our observations and successful treatment of two non-consanguineous Russian female patients diagnosed with nIHH. Prior to the referral to fertility clinic, each patient underwent about 10 years of hormone replacement therapy. In both patients, IVF/ET treatment was successful, with phenotypically healthy offspring delivered. In each patient, six candidate genes (*GnRH1, GnRHR, KISS1, KISS1R, TAC3, TAC3R*) were analyzed by sequencing. Patient 1 was a compound heterozygote for mutations in gonadotropin-releasing hormone encoding gene (*GnRH1*) and in gene encoding for its receptor (*GnRHR*), while in Patient 2 *GnRHR* mutations were found in homozygous state. In both patients, the coding frame of *GnRHR* gene harbored missense-mutation Arg139His previously described as founder mutation in Polish and Brazilan patients (Beneduzzi D et al., 2014) and in some other unrelated patients across the globe (Laitinen EM et al, 2012).

## Subjects and methods

### Patients

Both patients were referred to infertility clinic due to primary amenorrhea in absence of eating disorders or strenuous exercises, and infertility. Karyotypes were 46, XX, with normal sense of smell. Both patients received hormone replacement therapy with 2 mg oestradiol hemihydrate and a combination of 2 mg oestradiol hemihydrate/10 mg dydrogesterone (Femoston 2/10 mg, Abbott, Netherlands). Other characteristics of the patients and the results of their MRI and ultrasound examinations are shown in Table 1.

**Table 1:** Anthropometric and clinical characteristics of nIHH patients and their partners for IVF with ICSI

Characteristics	Patient 1	Patient 2
Age at first visit to fertility clinic	28 years	31 years
Pubarche (spontaneous)	at 14 years	at 15 years
Heights	170 cm	156 cm
Weight	65 kg	53 kg
Breast development by Tanner scale	Stage V	Stage IV
Sense of odors	Normal	Normal
Karyotype	46, XX	46, XX
MRI of the pituitary	*Size:* 10 mm x 6 mm x16 mm. Signs of signal reduction in the right half of the posterior pituitary (4 mm x 3 mm x 2 mm), depicting a cyst.	*Size:* 3.5 mm x 5 mm x 9 mm showing signs of moderate pituitary hypoplasia
Vaginal ultrasound	*Uterus:* 27 mm x 43 mm x 30 mm *Endometrium:* 2.3 mm *Right ovary:* 18 mm x 16 mm with 4 follicles (6 to 8 mm). *Left ovary:* 17 mm x 18 mm with 5 follicles (6 to 7 mm). Moderate hypoplasia of the uterus and ovaries.	*Uterus:* 35 mm x 22 mm x30 mm *Endometrium:* 1.8 mm *Right ovary:* 21 mm x 18 mm with 3 follicles (3 to 5 mm) *Left ovary:* 17 mm x 18 mm with 5 follicles (6 to 7 mm). Moderate hypoplasia of the ovaries.
Partner characteristics for IVF with ICSI	*Ejaculate volume:* 2 ml *Sperm concentration:* 39x10^6^/ml, *Motile sperms:* 3 % *Normal forms by Kruger strict criteria:* 2 %.	*Ejaculate volume:* 5 ml *Sperm concentration:* 31x10^6^/ml *Motile sperms:* None *Normal forms by Kruger strict criteria:* 1 %.

### *In vitro* fertilization (IVF) with intracytoplasmic sperm injection (ICSI)

In partners of both patients, the spermograms showed insufficient sperm quality (Table 1), thus justifying *in vitro* fertilization (IVF) with intracytoplasmic sperm injection (ICSI).

In both patients, oocytes were obtained by standard techniques within 36 h after administration of ovulatory dose of hCG (10,000 IU, Pregnyl, MSD, Netherlands), by follicle aspiration with 19G needle under ultrasound control. Obtained cumulus-oocyte complexes were placed in culture media (Universal IVF Medium, Origio, Denmark) for 2 h, then moved to medium containing recombinant human hyaluronidase (ICSI Cumulase, Origio, Denmark) for one minute, following by relocation into culture medium. To estimate the quality of oocytes, their shape and the presence of first polar body (metaphase II) were assessed. Living sperm cells selected for ICSI were immobilized in a drop of 10 % polyvinylpyrrolidone then injected into oocyte located at position 6 or 12 o’clock.

### DNA sequencing

Genomic DNA was isolated from the blood of two nIHH patients using phenol-chloroform technique. Exonic and promoter sequences for genes GNRH1 (NG_016457.1), GNRHR (NG_009293.1), KISS1 (NG_032151.1), KISS1R (NG_008277.1), TAC3 (NG_021398.1) and TAC3R (NG_023344.1) were amplified by PCR using primers located outside of intron-exon borders (Additional file 1: Table S1), the resultant PCR products were purified with DyEx columns (QIAGEN) and bidirectionally sequenced on the ABI PRISM®310 Genetic Analyzer (Applied Biosystems). Sequence data were analyzed with Chromas (Technelysium Pty.) software version 1.51 and compared with the RefSeq sequences indicated above.

### Ethical approval

Ethical approval was obtained from the local Institutional Review Board at Department of Obstetrics and Gynecology, State Pediatric Medical University, St.-Petersburg, Russia.

## Results

### Clinical outcomes

Upon initial nIHH diagnosis, both patients received combined hormone replacement therapy. Table 2 lists endogenous hormone values before the onset of reproduction assistance treatments.

**Table 2:** Hormone values before the onset of reproduction assistance treatments

Hormone	Patient 1	Patient 2	
		1 attempt	2 attempt
FSH	0.1 IU/L	0.75 IU/L	0.3 IU/L
LH	0.1 IU/L	0.26 IU/L	0.1 IU/L
TSH	5.4 μIU/ml	1.34 μIU/ml	2.24 μIU/ml
Estradiol	12 pg/ml	12 pg/ml	NA
Prolactin	202 mIU/ml	350 mIU/ml	153.8 mIU/ml
Inhibin B	8.8 pg/ml	NA	NA
AMH	0.1 ng/ml	1.96 ng/ml	0.93 ng/ml

*FSH* follicle stimulating hormone, *LH* luteinizing hormone, *TSH* thyroid-stimulating hormone, *AMH* anti-mullerian hormone, *NA* not assayed

#### Patient 1

To correct the levels of TSH, patient received L-thyroxin supplementation till her levels of TSH were brought to 2.8 nIU/ml. In IVF cycle, she received injections of human menopausal gonadotropin (300 IU, Menopur, Ferring) for 11 days. After administration of human chorionic gonadotropin (Pregnyl, MSD) at a dose of 10000 IU, twelve oocytes of good quality were retrieved. After fertilization by standard ICSI and 3 days of culture, two eight-cells embryos were transferred to uterine cavity, while three remaining good quality embryos were frozen. One gestational sac was detected in uterine cavity three weeks later.

After typical course of pregnancy, patient had uneventful spontaneous delivery of a girl, at week 39, with birth weight of 3150 g and length of 51 cm, Apgar scores were 9/9.

#### Patient 2

Ovarian stimulation was initiated with 225 IU of human menopausal gonadotropin in daily injections for 13 days, followed by induction of ovulation by human chorionic gonadotropin (10000 IU). Five good quality oocytes were retrieved. After conventional ICSI, two blastocysts of good quality were transferred into uterine cavity, while two remaining embryos were frozen. After four weeks, a viable pregnancy was detected. At 9 weeks of gestation, this pregnancy was lost. The cytogenetic analysis of miscarried embryo showed normal karyotype 46, XX. Subsequent transfer of frozen embryos was not successful.

One year after first attempt, another ovarian stimulation was performed, with 300 IU of human menopausal gonadotropin accompanied by FSH and LH for 15 days. After total FSH dose of 4275 IU, three oocytes were retrieved. After ICSI fertilization, two good quality embryos (A8 and A6) were transferred in the uterus of the patient.

Early and mid-pregnancy course was typical. Patient went into preterm birth due to placental abruption at week 37. The delivery of a boy was by C-section. Weight of a newborn was 3000 g, length was 50 cm, and Apgar scores were 8/9.

### Genetic analysis of the probands

Bidirectional sequencing was performed for 6 loci (*GnRH, GnRHR, KISS1, KISS1R, TAC3* and *TAC3R*) to cover all exons and their borders. In both patients, the mutations in *GnRH* and *GnRHR* genes were found (Table 3). In Patient 1 genome, a compound heterozygosity state was detected, with one copy of the *GnRH1* gene exon 1 (g. G6757C; p. W16S) and one copy of *GnRHR* gene exon 3 (g. C20426T; p. T269M) mutated. Additionally, another mutation (g. G7167A; p. Arg139His) was identified in exon 1 of the *GnRHR* gene in the same patient.

**Table 3:** The genotypes of the patients and their family members as assessed by bidirectional sequencing

*Gene*	Family 1			Family 2		
	P1	P1’s mother	P1’s offspring (girl)	P2	P2’s mother	P2’s offspring (boy)
*GNRH1 exon 1*	g.6757 G > C rs6185 Heterozygous	wt	wt	wt	wt	wt
	g.6891 T > G rs2709608 Homozygous intronic variant	g.6891 T > G rs2709608 Homozygous intronic variant	na	g.6891 T > G rs2709608 Homozygous intronic variant	g.6891 T > G rs2709608 Homozygous intronic variant	na
*GNRHR exon 1*	g. 7167 G > A rs104893842 Heterozygous	g. 7167 G > A rs104893842 Heterozygous	wt	g. 7167 G > A rs104893842 Homozygous	g. 7167 G > A rs104893842 Heterozygous	g. 7167 G > A rs104893842 Heterozygous
*GNRHR exon 3*	g. 20426 C > T Heterozygous	wt	wt	wt	wt	wt

*P1* Patient 1, *P2* Patient 2, *wt* wild-type, *na* not assessed

In patient 2, homozygous mutation in exon1 of the *GnRHR* gene (g. G7167A; p. Arg139His) was detected along with another homozygous intronic substitution (g. T6891G).

No mutations were detected in exons or promoter areas of *KISS1, KISS1R, TAC3* and *TAC3R*.

**Fig. 1:**
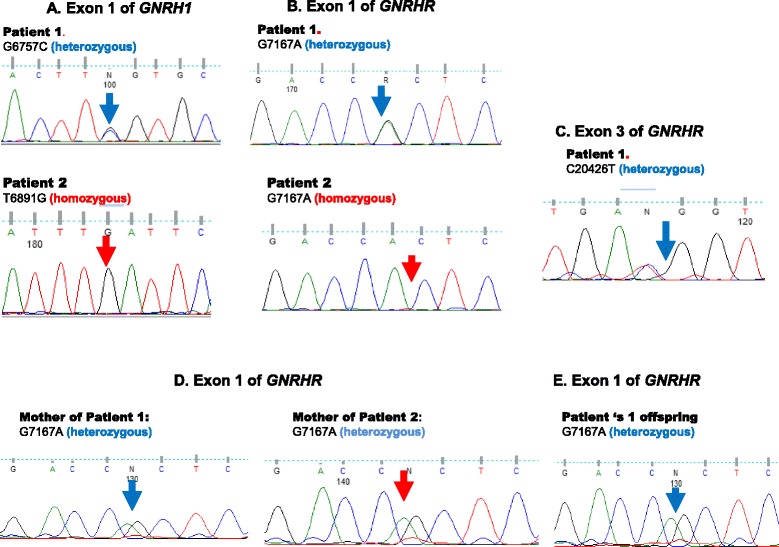
Sanger sequencing chromatograms of mutated loci in patients and their family members. **a** Sequencing chromatogram of the exon 1 of *GNRH1* gene in Patient 1 and Patient 2 genomes. **b** Sequencing chromatogram of the exon 1 of *GNRHR* gene in Patient 1 and Patient 2 genomes. **c** Sequencing chromatogram of the exon 3 of *GNRHR* gene in Patient 1 genomes. **d** Sequencing chromatogram of the exon 1 of *GNRHR* gene in the genomes of mothers of Patient 1 and Patient 2. **e** Sequencing chromatogram of the exon 1 of *GNRHR* gene in the genome of Patient 1 offspring

The mutations contributing to nIHH condition of the patients are summarized in Table 3 and Fig. 1 (a-c).

### Genetic analysis of the relatives of probands

Affected exons of *GnRH* and *GnRHR* genes were bidirectionaly sequenced in DNA samples in mothers and live offsprings of both probands.

In the genome of mother of Patient 1, heterozygous mutation of *GNRHR* exon 1 (g. 7167 G > A, rs104893842) was found, while *GNRHR* exon 3 was mutation free. No mutations were found in *GNRH1* exon 1. Mother of Patient 2 was also heterozygous by *GNRHR* exon 1 mutation (g. 7167 G > A, rs104893842). Mothers of both patients were also homozygous for g.6891 T > G variant in gene *GNRH1* (rs2709608).

The genotype of the offspring of patient 1 was wild-type for all informative loci, while the genome of the offspring of patient 2 carried heterozygous mutation g. 7167 G > A (rs104893842). In offspring, the state of the g.6891 T > G polymorphism in gene *GNRH1* (rs2709608) was not assessed. Observed genotypes are described in Table 3 and Fig. 1 (d, e).

## Discussion

In this study, the *GnRH1, GnRHR, KISS1, KISS1R, TAC3* and *TAC3R* genes were analyzed in two unrelated nIHH patients successfully treated for infertility. In both patients, Arg139His substitutions were identified within the receptor for hypothalamic gonadotropin-releasing hormone. In patient 1, this mutation was found in heterozygous state, while patient 2 was homozygous. Mutation p. Arg139His is located in a conserved DRS motif between the third transmembrane loop and the second intracellular loop. It completely inactivates the GnRH-binding activity of the receptor and prevents GnRH-induced stimulation of inositol phosphate accumulation *in vitro* [4].

In a recent study of Choi et al., R139H mutation was found to share a common founder core haplotype 220 kB in size. Interestingly, eight out of 15 carriers of this founder haplotype were of Polish origin [5]. In our study, both patients originated from Western region of Russia. In particular, Patient 1 and her mother were born in Pskov area that was under the winter-long siege by the Polish Army during the final stage of the Livonian War of 1558–1583. Hence, it is logical to conclude that p. Arg139His most likely was inherited from Polish founders. Also, it is tempting to speculate that R139 codon of *GNRHR* gene is a *hotspot* region for receptor-inactivating mutations, as the same codon was earlier found to contain another p.R139C mutation identified in two unrelated cases of Turkish origin [6, 7] and in Brazilian nIHH patients, where it was confirmed to associate with different founder haplotype [8].

Interestingly, *GNRHR* gene of Patient 1 also possessed a heterozygous T269M substitution that previously described in ClinVar as pathogenic in a nIHH patient of Asian/Indian origin. As the mother of patient 1 had no mutation in this locus, this substitution should be of either paternal origin or *de novo*. An absence of the mutation in Patient’s 1 husband and offspring genotypes suggests that Patient 1 genome is a cis-compound heterozygote for R139H and T269M mutations. In turn, that means that second allele of *GNRHR* in Patient 1 genome remains mutation free, and that one copy of wild type allele able to compensate for the deficiency of the receptor for GnRH binding only partially, at least in presence of pituitary deficiency and/or GnRH1 variant.

Indeed, the genome of Patient 1 had yet another heterozygous substitution, Trp16Ser [rs6185], in *GnRH1*. This variant was previously reported as polymorphism examined for its minor Ser^16^ allele association with decreased bone mineral density [9] and shorter disease-free survival in breast cancer patients [10]. Another recent study investigated this variant in polycystic ovarian syndrome (PCOS) and showed that homozygous or heterozygous carrier of Ser^16^ allele demonstrate somewhat more benign phenotype as compared with non-carriers, with lower follicle counts, as well as lower levels of testosterone, free androgen index and fasting insulin [11]. On the other hand, the presence of Ser^16^ variant had no influence on gonadotrophic hormone levels [11]. It would be tempting to speculate that Ser^16^ variant of hypothalamic gonadotropin-releasing hormone has lower potency than its wild-type variant, and could be a contributing factor to the reproductive phenotype of patient 1.

Importantly, reproductive interventions were successful in both nIHH patients despite the presence of GnRH resistance which is typical for women who have mutations in *GNRHR* [3, 12]. In nIHH patients, decreased circulating Anti-Mullerian Hormone (AMH) concentrations are not indicative of a decreased follicular reserve. An administration of human menopausal gonadotropin aids in overcoming ovarian insufficiency by inducing maturation of ovarian follicles. Women with nIHH should be informed that their infertility is treatable. In cases of homozygous founder mutations, mothers should be advised about the possibility that the chances for their offspring to inherit nIHH may increase if their partner comes from the same geographical region. However, the autosome recessive mode of inheritance along with non-life threatening, treatable nature of the nIHH condition make pre-implantation diagnostics unnecessary in the majority of cases.

## Conclusions

Polish founder mutation Arg139His in GnRHR was found in two nIHH patients originating from Western region of Russia. Common variant of GnRH-encoding gene, Trp16Ser, could possibly contribute to reproductive phenotypes in patients with heterozygous mutations of other GnRH signaling pathway genes. Women with nIHH should be informed that their infertility is treatable.

## Additional file

Additional file 1: Table S1. Primers for bidirectional Sanger sequencing in nIHH patients. (DOCX 15 kb)

## Abbreviations

AMH: Anti-Mullerian hormone
FSH: Follicle stimulating hormone
GnRH: Gonadotropin-releasing hormone
GnRHR: Gonadotropin-releasing hormone receptor
hCG: Human chorionic gonadotropin
ICSI: Intracytoplasmic sperm injection
IVF/ET: In Vitro Fertilization/Embryo Transfer
KS: Kallmann syndrome
LH: Luteinizing hormone
nIHH: Normosmic isolated hypogonadotropic hypogonadism
PCR: Polymerase chain reaction
TSH: Thyroid-stimulating hormone.
