# Supplementary material for: Mutations in gonadotropin-releasing hormone signaling pathway in two nIHH patients with successful pregnancy outcomes
Source: Reprod Biol Endocrinol. 2016 Aug 20;14:48. doi: 10.1186/s12958-016-0183-8 (PMC4992333; doi:10.1186/s12958-016-0183-8)
Supplement: Additional file 1: Table S1. — Primers for bidirectional Sanger sequencing in nIHH patients. (DOCX 15 kb) [file 12958_2016_183_MOESM1_ESM.docx]

Table S1. Primers for bidirectional Sanger sequencing in nIHH patients

| PRIMER | SEQUENCE |
| --- | --- |
| GNRH EXON 1 | Forward ATACTCAACCTTGTCTGGATC  Reverse GACGTGCTGTCTCACACTTG |
| GNRH EXON 2 | Forward AAGTCCCTATGCTAATCCTGC  Reverse CTGGAGTGGAGTAGTCTAGG |
| GNRH EXON 3 | Forward CCTAGCACTAACTAGAGCACA  Reverse GGCTGTTCTGCTTGTGGGTA |
| GNRHR EXON 1 (Part 1) | Forward ATGCACCAGAGACACAAGGC  Reverse TCGTGATAGCCAGGGAGCG |
| GNRHR EXON 1 (Part 2) | Forward ATCTGACCTTAGCCAACCTGT  Reverse AGGTAACAGAACAGAGCCAGA |
| GNRHR EXON 2 | Forward CATGTTAGAAGGCTAGCAGAG  Reverse TCACAATTTGTACTTGCTGCC |
| GNRHR EXON 3 | Forward TGAATTAGTGATGCTGTCTTCC  Reverse AATAATTGAGGCTCTGAAGAC |
| KISS1 EXON 1 | Forward TCTAGGACCTGCCTCTTCTC  Reverse GTTGCTGACGTAGAGCAGAG |
| KISS1 EXON 2 | Forward CCTCATCTTTCTGTGCCCTC  Reverse GCAAAAATGAGCCGCAGACC |
| KISS1R EXON 1 | Forward GCGCTCTCACTCCGACCTT  Reverse TTGCGACCTCTAGCCTCAG |
| KISS1R EXON 2 | Forward GCGTAATGTTTGAGATGTA  Reverse CAGTTCTAGGCTGCACCAC |
| KISS1R EXON 3 | Forward CCGTGTATGTGCCTGAGTGT  Reverse CGTGAAGGTGGTTAGACGA |
| KISS1R EXON 4 | Forward GTAGGTGAGTACAGCTCAG  Reverse CGTGAAGGTGGTTAGACGA |
| KISS1R EXON 5 | Forward CAGCCTTTCGTCTAACCACC  Reverse GTACACAGTGACAGTCAACT |
| TAC3 EXON 1 | Forward CAGGGCTTGGACAGTGGAG  Reverse CAGCTGTCCAGACAGGCAG |
| TAC3 EXON 2 | Forward GCTCTGTGTAGTGCTGGTGT  Reverse CTCACCGATTCACTAAGCTAT |
| TAC3 EXON 3 | Forward CGCTCTCATCACTTTCCAACT  Reverse GCTCTGGGCAAGTGGCAAG |
| TAC3 EXON 4 | Forward TAGGAGGAAGCTGGTGTGAG  Reverse CACGTGACTTCTGTCATACTC |
| TAC3 EXON 5 | Forward TAAGGTAGTCCTGTTGCAGCT  Reverse TCAGCTGCCGTCCATGATCC |
| TACR3 EXON 1 | Forward AGAACTTCAGAGGAGTCTCG  Reverse CTCTCTGTCTGTCCTCTCCT |
| TACR3 EXON 2 | Forward CCTAGACTATGCCATGATTAC  Reverse ATCCCACATAGACATCCTCAT |
| TACR3 EXON 3 | Forward TCACAGCAGCATACTTCTCG  Reverse ACATGCCATGACTAGATTGC |
| TACR3 EXON 4 | Forward GTCCGTATATTGCTTCACCAT  Reverse CCCATAGAAGAATCATCCAGT |
| TACR3 EXON 5 | Forward ATAAATTCTAAGAGTCTGGCT  Reverse TCAATTTGACCATAGCTGCCT |
